# Physical Activity, Cardiometabolic Health, and Functional Performance Across the Adult Lifespan: An Age-Stratified Real-World Primary Care Study

**DOI:** 10.3390/sports14070307

**Published:** 2026-07-20

**Authors:** Peter Marián Kalanin

**Affiliations:** 1 Institute of General Medicine, Faculty of Medicine, Pavol Jozef Šafárik University in Košice and MED-KAL, s.r.o., 040 01 Košice, Slovakia; peter.kalanin@upjs.sk; 2 Institute of Physical Education and Sport, Pavol Jozef Šafárik University in Košice, 040 11 Košice, Slovakia; 3 Department of Physiotherapy, Faculty of Health, Catholic University in Ružomberok, 034 01 Ružomberok, Slovakia

**Keywords:** physical activity, aging, adult lifespan, healthy aging, older-old adults, LDL cholesterol, functional performance, Timed Up and Go, primary care, real-world data, cardiometabolic risk, cross-sectional study

## Abstract

**Background**: Cardiometabolic risk and physical function are both strongly influenced by physical activity (PA) across the adult lifespan, yet it remains unclear whether this association is uniform across different stages of adulthood in everyday primary care settings, particularly among the oldest patients. **Objective**: Using a real-world primary care cohort stratified into four adult lifespan groups, we investigated how self-reported PA category was linked to LDL-C concentration and Timed Up and Go (TUG) performance, and formally tested whether these associations differed by age group. **Methods**: This cross-sectional analysis included 863 adult primary care patients, divided into four clinically meaningful lifespan groups: young adults (18–39 years, *n* = 277), middle-aged adults (40–59 years, *n* = 284), younger older adults (60–74 years, *n* = 166), and older-old adults (≥75 years, *n* = 136); PA was classified as low, moderate, or high per WHO recommendations. The primary outcomes were LDL-C and TUG. Between-group differences were evaluated with one-way ANOVA (Bonferroni-corrected post hoc tests) and age-specific multivariable linear regression adjusted for BMI, sex, arterial hypertension (AH), and diabetes mellitus (DM); a pooled PA × age-group interaction term formally tested for effect modification by age. **Results**: Baseline BMI, LDL-C, TUG, sex distribution, AH prevalence, and PA distribution were similar across the four groups, whereas DM prevalence differed modestly (*p* = 0.033). In every age group, higher PA categories were linked to significantly lower LDL-C (all *p* ≤ 0.006) and significantly better TUG times (all *p* < 0.001); PA was the only independent predictor shared by both outcomes across all groups in multivariable models (all *p* < 0.01). The standardized effect of PA on TUG was greatest among older-old adults (≥75 years: β = −1.609, 95% CI: −2.114 to −1.104, R^2^ = 0.260). Neither the PA–LDL-C nor the PA–TUG interaction with age group reached statistical significance (*p* = 0.571 and *p* = 0.349, respectively). **Conclusions**: Associations between PA and both cardiometabolic and functional performance outcomes were present across all four adult lifespan groups, including adults aged ≥75 years, with no statistically significant age modification detected in this sample. Given the exploratory nature of the interaction analysis and the limited size of the oldest-old subgroup, these results should be regarded as hypothesis-generating rather than conclusive evidence of age-invariance. The pattern seen among older-old adults (≥75 years) reinforces the clinical value of routine PA assessment for evaluating functional health in advanced age. Taken together, these results point to PA assessment as a candidate age-independent component of cardiometabolic and functional health evaluation across the adult lifespan in primary care, a possibility that larger, adequately powered samples should confirm.

## 1. Introduction

Habitual engagement in PA is consistently linked to better cardiometabolic health and physical functioning across the lifespan [1,2]. Considerable evidence links greater habitual PA with more favorable lipid profiles, lower cardiovascular risk, and better functional capacity [3,4,5]; routine PA assessment in primary care may therefore give clinicians practical insight into both cardiometabolic and functional status across varied patient populations.

Age is among the most influential determinants of cardiometabolic risk and functional capacity alike, with dyslipidemia, cardiovascular disease, and functional decline all growing more prevalent with advancing years [6,7]. Much of the existing PA literature nonetheless treats older adults (typically defined as ≥60 or ≥65 years) as a single, homogeneous category, overlooking substantial physiological and functional differences that distinguish younger older adults (60–74 years) from the older-old (≥75 years) [8]. This oldest group faces the highest risk of functional decline, falls, and cardiometabolic comorbidity, making it an especially important target for PA screening.

Determining whether the PA–outcome relationship is preserved across the full adult lifespan, including among the older-old, has direct clinical relevance. If such associations remain stable from early adulthood through advanced age, PA assessment could serve as a single, age-independent screening tool in primary care; conversely, evidence of substantial age-related variation would instead favor age-tailored PA screening strategies.

This same cohort has previously yielded separate reports on associations between self-reported PA and LDL-C [9], arterial hypertension [10], BMI-stratified healthy aging and functional outcomes [11], and sex-specific outcomes [12]; none of these earlier analyses, however, formally tested whether such associations differ across four distinct adult lifespan groups or specifically examined their preservation among adults aged ≥75 years. Additional companion analyses from the same cohort are currently undergoing the publication process.

Building on this earlier work, the present study examined associations between self-reported PA category and both LDL-C concentration and TUG performance across four adult lifespan groups, with formal testing of whether age group modifies these associations. We hypothesized that PA would relate similarly to both outcomes across age groups, without significant effect modification by age, extending the clinical rationale for PA assessment to the oldest primary care patients.

## 2. Materials and Methods

### 2.1. Study Design and Setting

**Figure 1:**
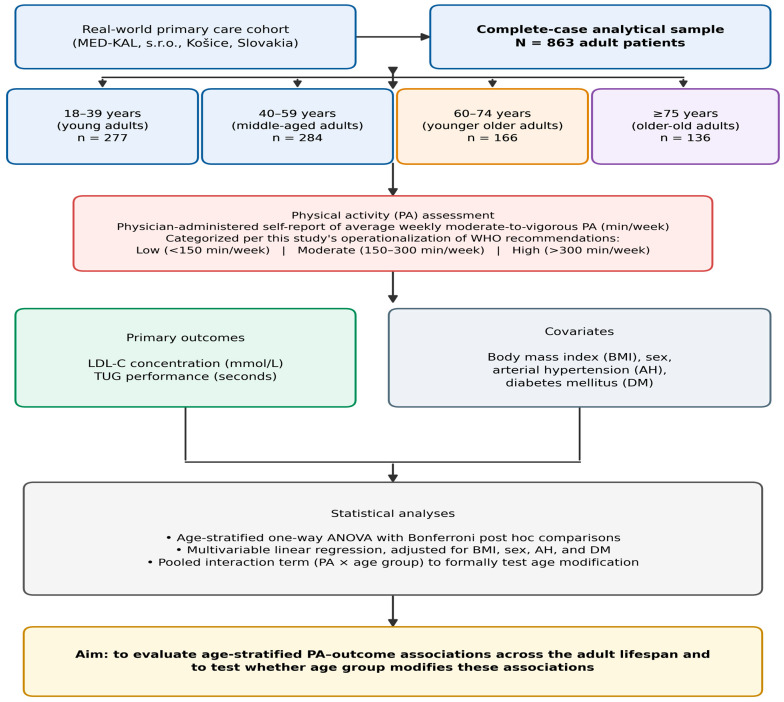
Study design and analytical framework. A total of 863 adult primary care patients meeting predefined complete-case inclusion criteria were stratified into four clinically meaningful adult lifespan groups: 18–39 years (*n* = 277), 40–59 years (*n* = 284), 60–74 years (*n* = 166), and ≥75 years (*n* = 136). PA was assessed via physician-administered self-report and classified as low, moderate, or high per this study’s operationalization of World Health Organization PA recommendations. Primary outcomes were LDL-C concentration and TUG performance, analyzed through age-stratified one-way ANOVA, multivariable linear regression, and pooled interaction analyses (PA × age group), adjusted for BMI, sex, AH, and DM.

This cross-sectional observational analysis drew on routinely collected clinical data from a primary care setting in Slovakia, with data collection spanning February 2021 to May 2026. Because recruitment was rolling, each patient contributed a single cross-sectional assessment at the time of their own routine visit rather than all participants being evaluated at one common time point; potential temporal shifts in PA guidelines, LDL-C assay calibration, and clinical practice over this period—including possible pandemic-related disruption to routine care in 2021—cannot be excluded and are noted as a limitation. Reporting followed the Strengthening the Reporting of Observational Studies in Epidemiology (STROBE) guidelines [13]. This analysis presents four-group age-stratified findings drawn from the same primary care cohort described in earlier publications [9,10,11,12]; the overall study design and analytical framework are summarized in Figure 1.

### 2.2. Study Population

The analytic sample consisted of 863 adults with complete records for PA category, LDL-C, TUG performance, and core clinical covariates. Eligibility required age ≥ 18 years, an available PA classification, LDL-C and TUG measurements, and complete core clinical data; patients missing any of these were excluded. Selection was independent of age group, PA level, or cardiometabolic risk status. Since the analytical dataset was assembled retrospectively from routine electronic medical records under pre-specified complete-case criteria, a detailed screening log could not be produced. The reported sample (*n* = 863) therefore corresponds to all patients from the underlying primary care cohort described in earlier publications [9,10,11,12] who met the predefined inclusion criteria for this analysis.

### 2.3. Age Group Classification

Stratification used four clinically meaningful adult lifespan groups: young adults (18–39 years, *n* = 277), middle-aged adults (40–59 years, *n* = 284), younger older adults (60–74 years, *n* = 166), and older-old adults (≥75 years, *n* = 136). This scheme was intended to capture distinct physiological life stages and to allow for separate examination of the older-old segment relative to younger older adults. The chosen cut-points (39/40, 59/60, and 74/75 years) follow a common epidemiological convention distinguishing young, middle-aged, young-old, and old-old adults [14], reflecting this study’s own operational choice rather than an externally standardized classification.

### 2.4. Data Collection

Electronic medical records generated during routine care supplied the clinical data, which were anonymized prior to analysis. Variables extracted included age, sex, BMI, LDL-C concentration, and AH and DM status. AH was recorded when a documented diagnosis and/or ongoing antihypertensive therapy was present; DM was defined analogously, based on a documented diagnosis and/or ongoing glucose-lowering treatment. LDL-C was quantified by enzymatic colorimetric assay in certified laboratories.

### 2.5. Physical Activity Assessment

PA level was determined through physician-administered self-report obtained during routine primary care evaluation, in which patients estimated their average weekly duration of moderate-to-vigorous PA (min/week); total weekly PA volume was derived from this estimate. Following World Health Organization dose–response recommendations, PA was categorized as low (<150 min/week), moderate (150–300 min/week), or high (>300 min/week) [15]—reflecting this study’s own operationalization of WHO guidance rather than an official WHO-defined category. Recall bias and exposure misclassification inherent to self-reported measures should be considered when interpreting these findings.

### 2.6. Outcome Measures

LDL-C concentration (mmol/L) and TUG functional performance (seconds) served as the primary outcomes. TUG followed the standardized protocol of Podsiadlo and Richardson [16], validated for reliability across the adult lifespan, including older populations [17,18]: participants rose from a standard armed chair, walked 3 m at their usual pace, turned, returned, and sat down again. The treating physician recorded time to the nearest 0.1 s using a handheld digital stopwatch (Garmin Ltd.; headquartered in Olathe, KS, USA), with one timed trial obtained per patient. The same physician administered all TUG assessments using an identical standardized protocol throughout the study period.

### 2.7. Statistical Analysis

Baseline characteristics were compared across age groups using one-way ANOVA for continuous variables and chi-square tests for categorical variables. PA-category associations with LDL-C and TUG were examined within each age group using one-way ANOVA with Bonferroni post hoc correction. Separate multivariable linear regression models were fitted for each age group, entering PA as an ordinal variable (low = 1, moderate = 2, high = 3); LDL-C models were adjusted for BMI, sex, AH, and DM, while TUG models additionally included LDL-C. A PA × age-group interaction term was added to pooled models to formally test for age modification, with significance set at *p* < 0.05. Because Bonferroni correction was applied to post hoc pairwise comparisons and the analysis involved multiple age-stratified tests and models, the age-stratified findings are best interpreted as hypothesis-generating. All statistical analyses were performed using Python (version 3.11; Python Software Foundation, Wilmington, DE, USA) with the NumPy and SciPy libraries.

### 2.8. Ethical Considerations

This study followed the principles of the Declaration of Helsinki (2013 revision) and used exclusively fully anonymized retrospective clinical data collected during routine primary care practice, in compliance with the applicable Slovak legal framework and GDPR; full details are provided in the Institutional Review Board Statement.

## 3. Results

### 3.1. Baseline Characteristics

Table 1 presents baseline characteristics across the four age groups. BMI, LDL-C, TUG, sex distribution, AH prevalence, and PA distribution were statistically similar across groups (all *p* > 0.05), while DM prevalence differed modestly (*p* = 0.033), peaking in the 40–59 and ≥75 year groups. Despite the wide age range spanning 18 to 85 years, this well-balanced cardiometabolic profile across groups enabled a meaningful age-stratified comparison of PA–outcome associations.

### 3.2. LDL-C Across PA Categories by Age Group

**Figure 2:**
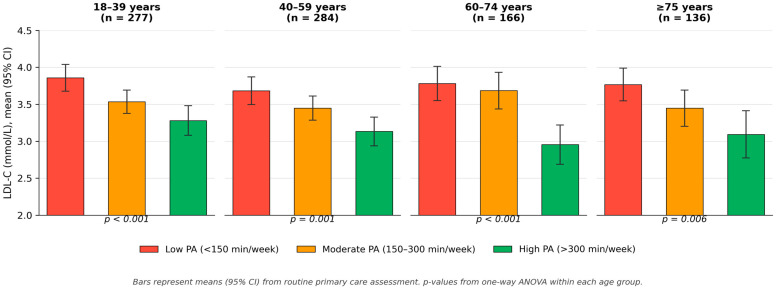
Mean LDL-C concentration by PA category within each adult lifespan group. Higher PA categories corresponded to lower LDL-C across all four groups (all *p* ≤ 0.006); error bars denote 95% confidence intervals. Displayed *p*-values derive from the overall one-way ANOVA within each age group, with Bonferroni-corrected pairwise post hoc comparisons. PA was categorized as low (<150 min/week), moderate (150–300 min/week), and high (>300 min/week), per this study’s operationalization of World Health Organization PA recommendations.

Table 2 and Figure 2 present LDL-C concentrations across PA categories by age group. Significantly lower LDL-C was observed with higher PA categories in each age group: 18–39 years (F = 8.00, *p* < 0.001), 40–59 years (F = 6.74, *p* = 0.001), 60–74 years (F = 9.66, *p* < 0.001), and ≥75 years (F = 5.26, *p* = 0.006). Post hoc testing showed significant high-versus-low PA differences in every age group (all *p* ≤ 0.003), with mean LDL-C differences between low- and high-PA groups of 0.58 mmol/L (18–39 years), 0.55 mmol/L (40–59 years), 0.82 mmol/L (60–74 years), and 0.68 mmol/L (≥75 years).

### 3.3. TUG Performance Across PA Categories by Age Group

**Figure 3:**
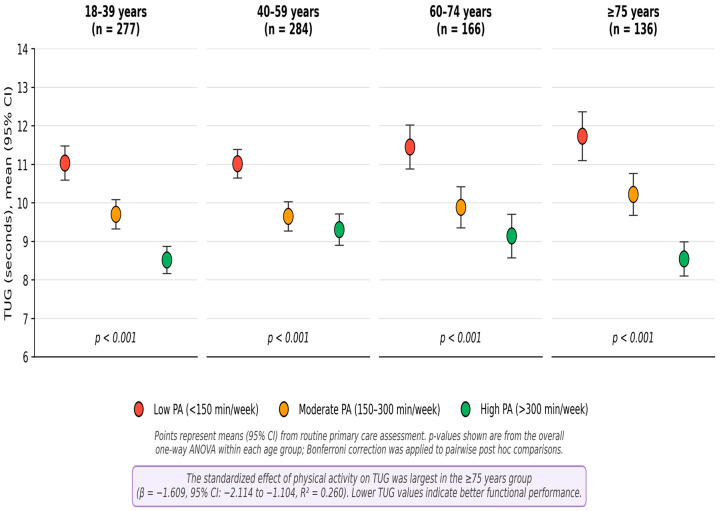
Mean TUG performance by PA category within each adult lifespan group. Better functional performance (lower TUG time) accompanied higher PA within every age group (all *p* < 0.001); error bars denote 95% confidence intervals. Displayed *p*-values derive from the overall one-way ANOVA within each age group, with Bonferroni-corrected pairwise post hoc comparisons. The strongest standardized PA–TUG association occurred among older-old adults (≥75 years; β = −1.609, R^2^ = 0.260).

Table 3 and Figure 3 present TUG performance across PA categories by age group. Significantly better TUG performance accompanied higher PA categories in every age group (all *p* < 0.001), and low-versus-high PA pairwise comparisons were significant throughout (all *p* < 0.001). Notably, in the older-old (≥75 years) group, all pairwise PA comparisons for TUG reached significance (low vs. moderate: *p* = 0.002; low vs. high: *p* < 0.001; moderate vs. high: *p* = 0.001), whereas the moderate-versus-high comparison in the 40–59 year group did not (*p* = 0.823)—suggesting the TUG gradient across PA categories may be steepest among older-old adults.

### 3.4. Multivariable Regression—LDL-C

Table 4 presents age-stratified multivariable regression results for LDL-C. PA category was the only consistently significant independent predictor of LDL-C after adjustment across all four groups: 18–39 years (β = −0.305, 95% CI: −0.451 to −0.159, *p* < 0.001), 40–59 years (β = −0.274, 95% CI: −0.419 to −0.128, *p* < 0.001), 60–74 years (β = −0.374, 95% CI: −0.566 to −0.183, *p* < 0.001), and ≥75 years (β = −0.336, 95% CI: −0.540 to −0.132, *p* = 0.002). Neither BMI, sex, AH, nor DM was independently associated with LDL-C in any group (all *p* > 0.05), and the PA × age-group interaction term was not significant (*p* = 0.571).

### 3.5. Multivariable Regression—TUG

Table 5 presents age-stratified multivariable regression results for TUG. PA category was the only consistently significant independent predictor of TUG performance in all four groups (all *p* < 0.001), with β coefficients of −1.312 (18–39 years), −0.865 (40–59 years), −1.261 (60–74 years), and −1.609 (≥75 years). The largest standardized PA–TUG effect occurred among older-old adults (≥75 years: standardized β = −0.499, R^2^ = 0.260). Neither BMI, LDL-C, sex, AH, nor DM was independently associated with TUG in any group (all *p* > 0.05), and the PA × age-group interaction term was not significant (*p* = 0.349).

## 4. Discussion

### 4.1. Principal Findings

In this real-world primary care cohort of 863 adults, higher self-reported PA category was linked to lower LDL-C and better TUG performance across all four lifespan groups, including those aged ≥75 years. PA was the only consistently significant independent predictor of both outcomes across all four age groups after multivariable adjustment. Formal interaction testing showed no significant age modification for either the PA–LDL-C (*p* = 0.571) or PA–TUG (*p* = 0.349) association. Because interaction effects generally require larger samples than main effects for reliable detection, and because the smallest age-by-PA subgroup in this study numbered only 25 participants, this non-significant interaction should be read as an absence of detected age modification in this sample rather than definitive proof of equivalence across age groups.

Particularly notable clinically is the pattern seen among older-old adults (≥75 years). This group showed the largest standardized β coefficient for PA on TUG (β = −1.609, standardized β = −0.499, R^2^ = 0.260), and every pairwise PA comparison for TUG reached significance—including moderate versus high PA (*p* = 0.001), which was non-significant in the 40–59 year group. This points to a TUG gradient across PA categories that may be steepest among older-old adults in primary care, reinforcing the relevance of PA assessment and promotion in advanced age.

Separating older adults into 60–74 and ≥75 year subgroups, rather than pooling them into a single ≥60 category, added clinical insight: PA–TUG associations were robust in both older groups, though somewhat stronger among the older-old (≥75 years). The LDL-C analysis additionally showed the lowest mean LDL-C across all age groups in the high-PA 60–74 year subgroup (2.96 ± 0.81 mmol/L), suggesting the lipid benefit of a high PA may be most pronounced among younger older adults.

### 4.2. Comparison with the Existing Literature

These results align broadly with existing evidence linking PA to favorable lipid profiles and functional performance across age groups [3,4,19], and with recent evidence that PA favorably affects multiple cardiometabolic risk factors including LDL-C, blood pressure, and glycemic control [20,21,22]. Prior work has generally supported associations between PA and favorable lipid profiles across adulthood, as well as better TUG performance among older populations [5,23]; the present findings extend this evidence to a real-world primary care population spanning four distinct lifespan groups, including the often-underrepresented oldest-old. The broader literature on sex-specific associations indicates a cardiovascular benefit from PA in both men and women, though the magnitude may vary by sex [24]. PA has also been shown to attenuate hypertension-related functional impairment among older adults [25], while reduced PA has been associated with poorer balance and gait among adults with type 2 diabetes [26]—patterns consistent with the covariate structure used here. The age-stratified interaction approach applied in this study parallels recent work on age × BMI interactions in TUG-based fall-risk assessment [27], underscoring the value of stratified over pooled analyses in heterogeneous older populations. The observed 0.55–0.82 mmol/L LDL-C differences between low- and high-PA groups across age strata may carry clinical relevance, comparable to effects reported for lifestyle interventions [28].

That PA emerged as the only consistently significant independent predictor of TUG, with the largest effect size among older-old adults (≥75 years), aligns with evidence that habitual PA strongly determines functional capacity and gait performance in advanced age, independent of conventional cardiometabolic risk factors [7,8]. This reinforces the broader literature supporting PA screening and promotion among older-old primary care populations to help prevent functional decline and falls. Because older adults commonly report specific, identifiable barriers to PA—including health concerns, fear of falling, and low motivation [29]—routine PA assessment in primary care could also serve as a natural entry point for identifying and addressing these barriers.

### 4.3. Clinical and Public Health Implications

That PA-associated differences in functional performance persist among older-old adults is clinically important, given that this group faces the greatest risk of mobility limitation, falls, and loss of independence [30]. The consistency of PA–outcome associations observed across all four adult lifespan groups—from young adults through the older-old—suggests routine PA assessment could function as a potentially age-independent component of cardiometabolic and functional health evaluation in primary care, pending confirmation in larger prospective studies. The persistence of PA-associated TUG differences among older-old adults specifically underscores the value of routine PA assessment for functional health evaluation even in advanced age.

These results suggest clinicians might consider incorporating simple PA screening into functional health assessment regardless of patient age, including among older-old primary care patients. That PA was the most consistent independent predictor of both LDL-C and TUG across all four lifespan groups—outperforming BMI, sex, AH, and DM—further highlights its potential value as a simple, practical screening indicator across the adult lifespan. Given this study’s observational, cross-sectional, single-site design, these implications should be viewed as hypothesis-generating; routine PA assessment may nonetheless represent a promising, low-cost, scalable approach for identifying adults at elevated cardiometabolic and functional risk across the lifespan, pending confirmation in prospective, multi-site research.

### 4.4. Limitations

Several limitations warrant acknowledgment. First, causal inference is precluded by the cross-sectional design, and reverse causation cannot be excluded. Second, PA relied on physician-administered self-report rather than a standardized validated questionnaire or objective monitoring such as accelerometry [31], introducing potential recall bias and exposure misclassification. Third, despite adjustment for BMI, sex, AH, and DM, residual confounding from unmeasured factors—including lipid- or function-affecting medications, musculoskeletal conditions, frailty, nutritional status, and socioeconomic circumstances—cannot be ruled out. Formal regression diagnostics (linearity, homoscedasticity, residual normality, and multicollinearity) were not separately reported; given the modest sample sizes within some age-by-PA subgroups, particularly the ≥75-year group (*n* = 136), such diagnostics would, in any case, have had limited precision, an additional acknowledged limitation. Relatedly, the rolling data collection window (February 2021–May 2026) introduces potential confounding from temporal shifts in clinical practice, PA guidance, or assay calibration not captured by the regression covariates. Fourth, generalizability may be limited by the single-site design within one Slovak primary care practice. Although the ≥75-year group was smaller than the younger strata, PA-category distribution remained adequate for exploratory age-stratified analysis; the findings in this subgroup nonetheless warrant cautious interpretation given the reduced sample size. The age-group boundaries used here may also diverge from those used in other epidemiological studies, potentially limiting cross-cohort comparability. Fifth, because the analytical sample derived from routine electronic medical records under pre-specified complete-case criteria, no formal patient-by-patient screening and exclusion log was maintained; consequently, the extent and pattern of missingness in the source cohort, and its potential to bias age-stratified comparisons, cannot be fully characterized—consistent with broader challenges in ensuring fitness-for-purpose of real-world data from routine electronic health records [32]. Sixth, a plausible alternative explanation for the flat PA–outcome pattern across age groups involves selection tied to inclusion criteria: because enrollment required both a recorded PA classification and an in-clinic TUG assessment, the oldest-old patients captured here likely represent a relatively high-functioning, non-frail subset of that age group, since frail, homebound, or cognitively impaired patients would be less likely to have both measures recorded. This “healthy attender” selection could itself explain the absence of a detected age interaction, regardless of whether a true interaction exists in the broader ≥75-year population, and should inform interpretation of the observed cross-group consistency; such selection bias is a well-documented methodological challenge in observational research involving older adults [33]. Seventh, although family-wise correction for multiple comparisons was not applied across the full set of age-stratified tests, the directional consistency of associations across analyses mitigates, without eliminating, the risk of false-positive findings arising from multiplicity.

### 4.5. Future Directions

Future research should employ longitudinal designs and objective PA measurement to clarify temporal and causal relationships between PA and cardiometabolic/functional outcomes across adult lifespan groups, with particular attention to the oldest-old segment. Incorporating frailty indices, objective gait analysis, comprehensive medication data, and direct measures of muscle strength and functional reserve would offer a more complete picture of PA–health relationships in advanced age.

## 5. Conclusions

Across four adult lifespan groups—young adults (18–39 years), middle-aged adults (40–59 years), younger older adults (60–74 years), and older-old adults (≥75 years)—this real-world primary care cohort demonstrated that higher self-reported PA was linked to lower LDL-C and better TUG performance. PA category remained the only consistently significant independent predictor of both outcomes across all age groups after multivariable adjustment, with no statistically significant age modification detected. The largest standardized PA–TUG effect occurred among older-old adults (≥75 years), reinforcing the particular clinical relevance of PA assessment for functional health evaluation in advanced age. These findings suggest PA assessment may function as a potentially age-independent component of cardiometabolic and functional health evaluation across the adult lifespan in primary care, pending confirmation in larger, longitudinal research incorporating objective PA measurement.

## Figures and Tables

**Table 1:** Baseline demographic and clinical characteristics by age group. Continuous variables compared using one-way ANOVA. Categorical variables compared using chi-square test. Abbreviations: AH, arterial hypertension; BMI, body mass index; DM, diabetes mellitus; LDL-C, low-density lipoprotein cholesterol; PA, physical activity; SD, standard deviation; TUG, Timed Up and Go. Given the right-skewed distribution of TUG, median (IQR) is additionally reported below the mean ± SD in each cell; the range for age is reported below the mean ± SD in the Total column.

Variable	18–39 y (*n* = 277)	40–59 y (*n* = 284)	60–74 y (*n* = 166)	≥75 y (*n* = 136)	Total (*n* = 863)	*p*
Age (years), mean ± SD	28.6 ± 6.3	49.3 ± 6.1	66.8 ± 4.3	79.9 ± 3.3	50.9 ± 19.3 range 18–85	<0.001
Female sex, *n* (%)	148 (53.4%)	144 (50.7%)	74 (44.6%)	73 (53.7%)	439 (50.9%)	0.285
BMI (kg/m^2^), mean ± SD	27.5 ± 4.5	28.0 ± 4.8	27.8 ± 4.3	27.2 ± 4.9	27.7 ± 4.6	0.251
LDL-C (mmol/L), mean ± SD	3.62 ± 0.91	3.48 ± 0.93	3.57 ± 1.00	3.51 ± 0.90	3.54 ± 0.94	0.343
TUG (seconds), mean ± SD	10.01 ± 2.28 median 9.80 (8.30–11.50)	10.12 ± 2.10 median 10.00 (8.60–11.60)	10.40 ± 2.37 median 10.30 (8.60–11.90)	10.50 ± 2.37 median 10.40 (8.67–12.33)	10.20 ± 2.26 median 10.10 (8.50–11.70)	0.116
AH, *n* (%)	150 (54.2%)	155 (54.6%)	100 (60.2%)	76 (55.9%)	481 (55.7%)	0.614
DM, *n* (%)	40 (14.4%)	59 (20.8%)	20 (12.0%)	29 (21.3%)	148 (17.2%)	0.033
Low PA, *n* (%)	108 (39.0%)	111 (39.1%)	71 (42.8%)	53 (39.0%)	343 (39.7%)	0.801
Moderate PA, *n* (%)	120 (43.3%)	118 (41.5%)	59 (35.5%)	58 (42.6%)	355 (41.1%)	
High PA, *n* (%)	49 (17.7%)	55 (19.4%)	36 (21.7%)	25 (18.4%)	165 (19.1%)	

**Table 2:** LDL-C concentrations across PA categories by age group. Post hoc comparisons performed using Bonferroni correction. Bold ANOVA *p*-values indicate statistical significance at *p* < 0.05. Abbreviations: LDL-C, low-density lipoprotein cholesterol; PA, physical activity; SD, standard deviation.

PA Category	18–39 y LDL-C Mean ± SD	40–59 y LDL-C Mean ± SD	60–74 y LDL-C Mean ± SD	≥75 y LDL-C Mean ± SD	ANOVA *p* Within Group
Low PA	3.86 ± 0.96	3.68 ± 1.01	3.78 ± 1.00	3.77 ± 0.82	
Moderate PA	3.53 ± 0.89	3.45 ± 0.90	3.69 ± 0.97	3.45 ± 0.95	
High PA	3.28 ± 0.72	3.13 ± 0.73	2.96 ± 0.81	3.09 ± 0.81	
**ANOVA *p***	*p* < 0.001 (F = 8.00)	*p* = 0.001 (F = 6.74)	*p* < 0.001 (F = 9.66)	*p* = 0.006 (F = 5.26)	All *p* < 0.05

**Table 3:** TUG performance across PA categories by age group. Post hoc comparisons performed using Bonferroni correction. Bold ANOVA *p*-values indicate statistical significance at *p* < 0.05. Abbreviations: PA, physical activity; SD, standard deviation; TUG, Timed Up and Go.

PA Category	18–39 y TUG Mean ± SD (s)	40–59 y TUG Mean ± SD (s)	60–74 y TUG Mean ± SD (s)	≥75 y TUG Mean ± SD (s)	ANOVA *p* Within Group
Low PA	11.04 ± 2.34	11.02 ± 2.01	11.45 ± 2.44	11.73 ± 2.36	
Moderate PA	9.71 ± 2.12	9.65 ± 2.11	9.89 ± 2.08	10.22 ± 2.12	
High PA	8.52 ± 1.26	9.31 ± 1.55	9.14 ± 1.73	8.55 ± 1.13	
**ANOVA *p***	*p* < 0.001 (F = 26.83)	*p* < 0.001 (F = 19.44)	*p* < 0.001 (F = 15.94)	*p* < 0.001 (F = 20.80)	All *p* < 0.001

**Table 4:** Age-stratified multivariable linear regression for LDL-C. PA category entered as ordinal variable (low = 1; moderate = 2; high = 3). ** *p* < 0.01; *** *p* < 0.001. Abbreviations: AH, arterial hypertension; BMI, body mass index; CI, confidence interval; DM, diabetes mellitus; LDL-C, low-density lipoprotein cholesterol; PA, physical activity.

Variable	18–39 β	18–39 95% CI	40–59 β	40–59 95% CI	60–74 β	60–74 95% CI	≥75 β	≥75 95% CI
PA (per increase) ***	−0.305	−0.451 to −0.159	−0.274	−0.419 to −0.128	−0.374	−0.566 to −0.183	−0.336 **	−0.540 to −0.132
BMI	−0.009	−0.033 to 0.014	0.006	−0.016 to 0.028	−0.002	−0.037 to 0.033	−0.015	−0.046 to 0.016
Sex (male)	−0.111	−0.322 to 0.100	0.036	−0.180 to 0.251	−0.137	−0.434 to 0.160	0.115	−0.186 to 0.416
AH	0.099	−0.113 to 0.311	0.041	−0.175 to 0.258	0.086	−0.219 to 0.390	−0.078	−0.379 to 0.222
DM	−0.066	−0.367 to 0.235	−0.010	−0.277 to 0.256	−0.206	−0.660 to 0.248	0.199	−0.166 to 0.564
R^2^	0.064		0.047		0.096		0.097	
Interaction PA × age group p (LDL)	*p* = 0.571							

**Table 5:** Age-stratified multivariable linear regression for TUG performance. PA category entered as ordinal variable. *** *p* < 0.001. Abbreviations: AH, arterial hypertension; BMI, body mass index; CI, confidence interval; DM, diabetes mellitus; LDL-C, low-density lipoprotein cholesterol; PA, physical activity; TUG, Timed Up and Go.

Variable	18–39 β	18–39 95% CI	40–59 β	40–59 95% CI	60–74 β	60–74 95% CI	≥75 β	≥75 95% CI
PA (per increase) ***	−1.312	−1.663 to −0.960	−0.865	−1.188 to −0.542	−1.261	−1.716 to −0.807	−1.609 ***	−2.114 to −1.104
BMI	−0.003	−0.058 to 0.052	0.005	−0.043 to 0.054	0.018	−0.061 to 0.097	−0.059	−0.134 to 0.015
LDL-C	−0.075	−0.353 to 0.203	0.177	−0.078 to 0.431	−0.164	−0.516 to 0.188	−0.026	−0.439 to 0.387
Sex (male)	0.188	−0.306 to 0.682	−0.191	−0.656 to 0.275	−0.335	−1.012 to 0.341	−0.026	−0.744 to 0.692
AH	0.434	−0.062 to 0.930	−0.194	−0.663 to 0.275	−0.202	−0.894 to 0.490	0.268	−0.449 to 0.984
DM	0.590	−0.113 to 1.293	0.334	−0.242 to 0.910	−0.267	−1.300 to 0.767	0.379	−0.493 to 1.251
R^2^	0.182		0.121		0.169		0.260	
Interaction PA × age group p (TUG)	*p* = 0.349							

## Data Availability

The datasets generated and/or analyzed during the current study are not publicly available due to privacy and data protection restrictions, as the dataset is derived from a real-world primary care patient cohort, but are available from the corresponding author on reasonable request, subject to applicable ethical and legal requirements.
